# Age- and gender-specific improvements on health-related quality of life (HRQoL) and cardiovascular fitness in healthy and sporting inactive older adults using a multidimensional exercise program

**DOI:** 10.3389/fspor.2025.1641115

**Published:** 2025-09-25

**Authors:** Anneke Schumacher, David Rahil, Marlene Krumpolt, Alexander Prinz, Lucas Sannemann, Kerstin Witte

**Affiliations:** Department of Sport Science, Otto-von-Guericke-University Magdeburg, Magdeburg, Germany

**Keywords:** subjective perception of physical well-being, motor performance, older adults 60+, submaximal cycle ergometry test, popular sports

## Abstract

**Introduction:**

Various training regimes in older adults yield differing effects on subjective well-being and physical performance, influenced by age, gender, and individual health status, with noted discrepancies between perceived well-being and actual performance.

**Methods:**

This study investigated age- and gender-specific effects of a 24-week multidimensional training program—including popular sports—on 81 sporting inactive, healthy older adults (60–74 years; f = 53, m = 28). Cardiovascular fitness was assessed via Physical Working Capacity 130 and health-related quality of life via ShortForm 36.

**Results:**

Women and participants aged 65–69 years showed the greatest subjective health gains and improvements in cardiovascular fitness (*p* < .001, d = .45). However, Spearman correlations indicated no significant association between subjective Health and objective fitness at baseline (*p* = .149) or post-intervention (*p* = .321), nor between changes in performance (total group *p* = .601; age- and sex-stratified change correlations all *p* > .191), indicating that women with lower baseline performance did not show systematically larger subjective gains.

**Discussion:**

The program thus offers an effective, non-traditional option to enhance physical fitness and health perception in older adults.

## 1 Introduction

The aging of Western societies, including Germany, is leading to a steadily growing proportion of older adults (65+), posing substantial economic challenges for healthcare systems (1, 2). Promoting healthy aging through lifestyle-related measures—such as regular physical activity, balanced nutrition, and social participation—is therefore a public health priority (3, 4). Among these factors, physical activity plays a central role, particularly in improving cardiorespiratory fitness and reducing the risk of chronic disease and premature mortality (5). Beyond physical health, physical activity is also associated with improved psychosocial well-being and enhanced health-related quality of life (6, 7).

Despite these benefits, there is a persistent discrepancy between objectively measured physical performance and subjective health perceptions, especially in older adults (8–10). For instance, individuals with high physical capacity may still report low health-related quality of life (HRQoL), which has been linked to a higher risk of falls (11). This disconnect is particularly pronounced among women, who generally report lower HRQoL than men across all age groups despite comparable or superior objective health indicators (11–15). These gender differences in perception, risk profiles, and training preferences highlight the need for gender-sensitive interventions (16), as evidence shows that men typically achieve greater absolute strength gains, whereas women often display relatively larger improvements in endurance and functional parameters—differences rooted in multifactorial physiological mechanisms and reinforcing the importance of sex-specific program design and dose adjustment (17).

To maintain or improve subjective well-being, low-intensity and multimodal training programs have shown promising effects, especially when designed to meet individual needs and preferences (6, 7, 18). However, while low-threshold training approaches like multicomponent training programs (MTPs) and resistance training (RT) are widely applied, existing research lacks clarity on how sex and age moderate their effects (17, 19, 20). In particular, inconsistencies in findings on concurrent training (CT) efficacy and the varying responses observed in different age groups (e.g., 50–65 vs. 66–73 years) point to a critical gap in personalized exercise prescriptions for older adults (20). Furthermore, recent studies have shown that health improvements gained from training are highly sensitive to detraining, especially within the first three months of inactivity (21–23).

Structured MTPs and RT programs have demonstrated broad benefits for cardiovascular, metabolic, and functional health in older adults. For example, supervised programs led to significant improvements in lipid profiles, strength, and blood pressure regulation, while detraining caused rapid declines in these parameters (21–23). Additionally, specific RT modalities (e.g., power or strength-endurance training) can improve balance, physical activity levels, and aspects of QoL in older women (24). Nevertheless, the relationship between physical improvements and perceived well-being remains inconsistent. Several studies report weak or absent correlations between HRQoL (e.g., SF-36 scores) and objective fitness indicators such as the Physical Working Capacity 130 (PWC-130) (25–27), underscoring the need for interventions that better align physiological and psychological health outcomes.

Moreover, gender-specific adaptations to exercise must be better understood. Recent findings suggest that women may show relatively greater gains in strength despite men achieving higher absolute values, highlighting the importance of individualized, gender-sensitive approaches to training—a principle aligned with the emerging field of Precision Exercise Medicine (28).

This study aims to address these research gaps by evaluating the age- and gender-specific effects of a 24-week, multidimensional, low-threshold exercise program on the subjective well-being and physical performance of previously sporting inactive older adults. Unlike traditional mono-focused interventions, this program incorporates elements of popular recreational sports such as dancing, bowling, and karate to foster engagement through spontaneous movement, varied motor skills, and social interaction (29–31).

By investigating subgroup-specific responses, training adherence, and the alignment between objective and subjective health indicators, this study contributes to the development of sustainable, inclusive exercise strategies tailored to the diverse needs of aging populations. It also adds to the limited and inconsistent body of evidence on concurrent training effects in older adults, offering practical recommendations for scalable, evidence-based health promotion.

## 2 Materials and methods

### 2.1 Study design

Considering the process of implementation and the potential habituation to exercise, the study was designed as a pre-post trial comprising a 24-week sports program (32, 33). Various motor and health-related tests were administered at two measurement points [pre-test before start (T0) and post-test after 24 weeks (T1)]. The research protocol conformed to the principles of the Declaration of Helsinki and was approved by the Ethics Committee of the Otto-von-Guericke University Magdeburg (Germany) (3/22). Participants were informed in detail about the purpose of the study at the first meeting and provided written informed consent. Even during the coronavirus pandemic, sport could be continued under certain hygienic conditions.

### 2.2 Sample description

The present study—from February 2022 to February 2024—involved 154 older adults aged 60–82 years who did not regularly exercise and were physically healthy except for age-related illnesses. The sample was recruited through advertisements in the local newspaper and screened by telephone interview for exclusion criteria related to sporting activity and acute illness. To exclude very severe or acute functional limitations, such as those of heart attack or stroke patients, medical clearance was obtained from the participants. Due to the ethical guidelines of the funding agency, the National Association of Statutory Health Insurance Funds, all participants who met the inclusion criteria were included in the intervention group to help as many people as possible to lead a healthier and more active lifestyle. Of the 154 participants, data is available for 128 older adults, as 26 participants were unable to meet the 75% attendance requirement or had to drop out of the project for personal reasons. An *a priori* power analysis was conducted using G*Power (Version 3.1.9.7) for a t-test with dependent groups (*α* = .05, 1–*β* = .95, effect size f = 0.5), yielding a required total sample size of 44 participants and for Wilcoxon-test a total sample size of 35 participants.

### 2.3 Multidimensional exercise program

During the six-month exercise program, the participants practice twice a week for 90 min in fixed sports groups of a maximum of 20 participants, mainly in the gym of the University of Magdeburg, following a predetermined training plan for all activities, which was the same across all groups regarding the sports offered. Typical sports for older adults are increasingly pushed into the background and the focus is on a wide range of (popular) sports. The first session of the week is a fitness training led by licensed instructors and includes a combination of coordination, strength and endurance exercises, using the varying equipment of the gym (benches, mats) as well as small devices such as dumbbells, resistance bands, sticks or medicine balls. The second session of the week is an introduction class run by coaches from local sports clubs to offer their sports. Fifty percent of the exercise program therefore consisted of sports such as bowling, karate, yoga, soccer, or badminton. The aim is to introduce participants to as many sports as possible through basic elements and techniques or playful exercises of these sports to try them out. The advantage of this exercise concept is that many men who would not participate in typical health exercise programs participate in the sport and thus have a preventive effect on their health (29). As the project addresses at inactive older adults, it starts from a low threshold in terms of exercise norms and increases over time, using methods such as High Intensity Interval Training (HIIT) or game-based activities to strengthen endurance. Only speed exercises are excluded from the training to avoid injuries.

### 2.4 Instruments and procedure

As this study was sponsored and funded by statutory health insurance funds, and the incidence of cardiovascular disease has increased significantly in this region, it was important to analyze exercise capacity about cardiovascular parameters. In addition, the sample is characterized by an inactive sporting lifestyle, so a low-threshold step test on the bicycle ergometer, the PWC 130, was preferred. The SF-36 was used as a standardized method to record the subjective assessment of the participant's physical health.

#### 2.4.1 Motor test and outcome parameters

The PWC 130 (Physical Working Capacity) is a test designed to assess the physical performance of older people, in particular the submaximal endurance capacity based on the cardiovascular system (34). The WHO exercise protocol (25/25/2), pre-installed and automatically controlled in the cycle ergometer Cortex Bike M, was used because it is suitable for older untrained people (35, 36). All subjects start with 25 watts resistance at a speed of 70 rpm and must gradually increase the load by 25 watts every two minutes. Heart rate is recorded using a Polar Electro chest strap with a heart rate sensor both before the test (resting heart rate), at each step change and at the end of the difficulty level when the target heart rate of 130 bpm is exceeded. The test is completed when the target heart rate of 130 bpm has been exceeded for 30 s In addition to the wattage achieved and the heart rate (absolute performance), the subject's blood pressure is recorded for safety reasons. In order to make the results comparable, the calculation of Rost et al. (37) of the relative wattage to the target heart rate per kilogram of body weight is used. Standard values for women are lower than for men, as women have approximately 15%–20% less muscle mass in relation to their body weight.

#### 2.4.2 SF-36

The Short Form Questionnaire 36 modified (38) is used to measure the health related quality of life (HRQOL) and can be applied to normal population as well as to other groups of patients (39, 40). Its 36 items cover eight dimensions of subjective health: physical functioning, physical role functioning, bodily pain, general health, vitality, social functioning, emotional role functioning, and mental health are expressed with value ranges of a 0–100 scale. These subscales can be used to create a mental component score (MCS) or a physical component score (PCS), which was analyzed in this study. A higher value on the component scores comes in line with a better state of health.

### 2.5 Statistical data analysis

The PWC-130 and SF-36 data were analyzed using SPSS (version 28) (41). For the analysis of the SF-36, a syntax of the measurement instrument is available. At least 50% of the items on a subscale must be answered to obtain an assessment of subjective health perception. Subsequently, the raw scores of the component score are calculated and transformed into a percentage scale value from 0 to 100.

After checking the data for normal distribution, Mann–Whitney *U* and Wilcoxon tests were applied due to non-normally distributed data, the presence of outliers, and slightly unequal sample sizes to compare the group medians. In addition, Spearman's correlation was calculated between the relative performance from PWC 130 and the PCS of the SF-36. For further analysis, the sample was divided according to gender and age, with the age range in the age groups limited to five years (60–64 years, 65–69 years, 70–74 years and 75+). To account for multiple testing, the significance level was adjusted to *α* = .01. Significant differences were calculated using the effect size Pearson's *r*. A small effect is present from *r* ≥ 0.1, a moderate effect from *r* ≥ 0.3, and a large effect from *r* ≥ 0.5.

## 3 Results

### 3.1 Sample

**Figure 1:**
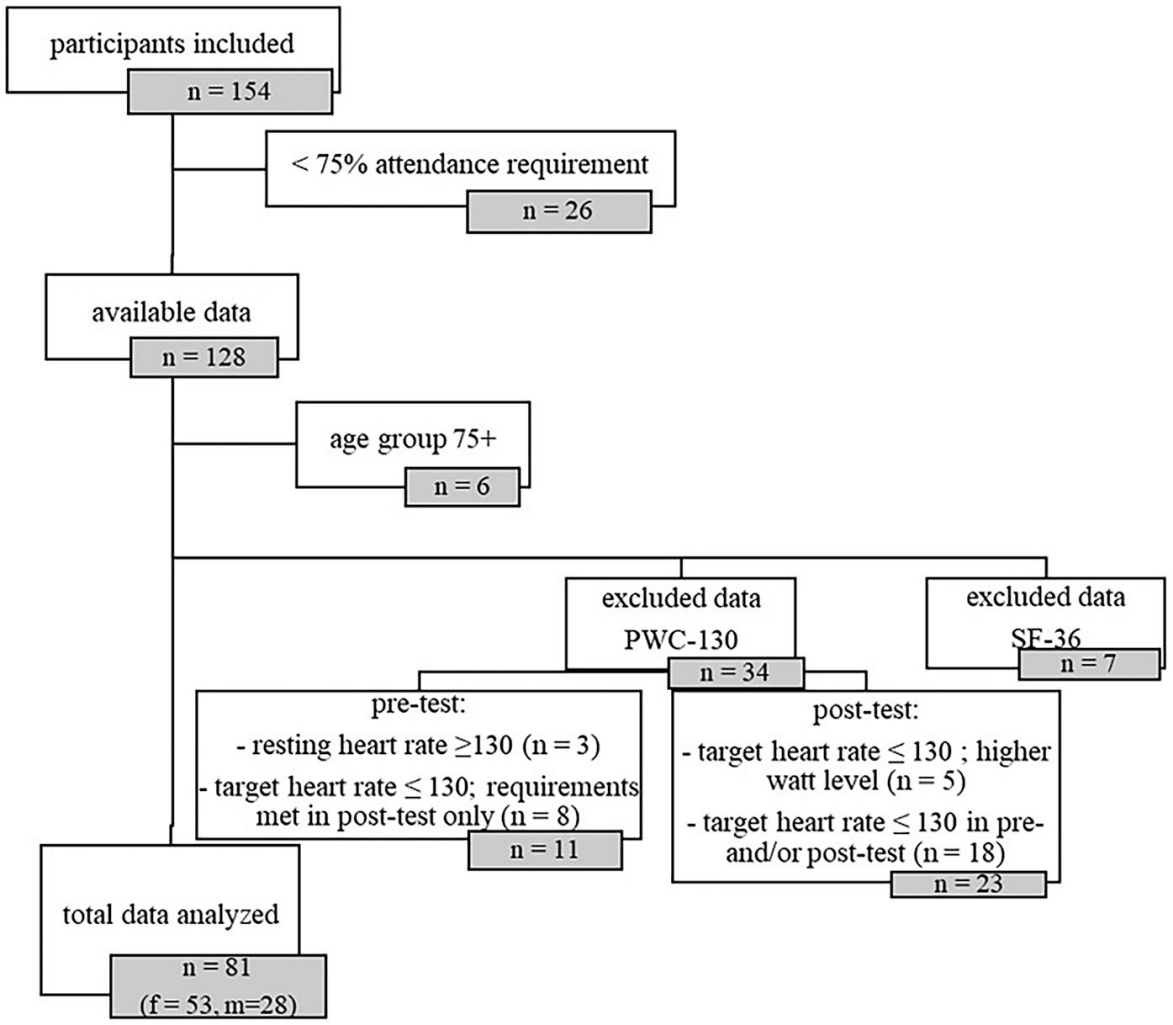
Trial-flow chart.

There were too few subjects in the 75+ age group (*n* = 6) for the results to be included in the overall analysis. Out of the 122 older adults data sets, approximately 28% of the results (*n* = 34) were excluded because the PWC-130 requirements were not met due to various stopping criteria: 3 participants had a resting heart rate of over 130 bpm on the pre-test before exercise, 18 participants did not meet the target heart rate of 130 bpm on either the pre-test or the post-test due to lack of strength or endurance performance, and a further 8 participants were only able to meet the exercise requirements on the post-test. 5 seniors did not achieve the target heart rate of 130 bpm on the post-test because they were physically unable to sustain the effort at a higher level of difficulty than on the pre-test. Data from an additional 7 participants are missing from the analysis because the SF-36 questionnaires were not completed correctly. In total the data of 81 participants (age 67 ± 3.3 years; f = 53, m = 28) were included in the analysis (see Figure 1).

Older adults are represented in the age groups 60–64 years with 22.2% (*n* = 18; m = 4; w = 14), 65–69 years with 51.9% (*n* = 42; m = 12; w = 30) and 70–74 years with 25.9% (*n* = 21; m = 12; w = 9). Due to the sample size, age groups were not further broken down by gender. From a sports biographical perspective, there is an even distribution of years of inactivity per age group: About 20% of participants stated that they had been inactive (a) since the start of the coronavirus pandemic, (b) for more than 5 years, (c) for more than 10 years, (d) for more than 20 years, or (e) that they had never been regularly active.

### 3.2 Outcome parameters

#### 3.2.1 PWC-130

In both the pre- and post-tests, the Mann–Whitney-*U*-Test showed that there are differences in the absolute performance of the PWC-130 with regard to gender and that men achieved a higher watt level (*p* < .001). As indicated in the methods section, this is typically due to the difference in muscle mass between men and women. The relative performance of the PWC-130 contained no significant difference in the gender comparison (pre-test: *p* = .153; post-test: *p* = .323). There are no significant differences between the three age groups on either the pre- or post-test neither in absolute nor in relative performance (pre-test: all *p* > .20, post-test: all *p* > .40). Regarding the multidimensional exercise program, a significant change over time was found for the entire sample and for women with a moderate effect (r = .39–.45). When stratified by age, none of the age groups reached the adjusted significance threshold of *α* = .01 for relative or absolute PWC-130 values (see Table 1).

**Table 1:** Absolute and relative performance results from PWC-130 baseline (T0) and post-test (T1).

Task		Absolute wattage [W]				Relative wattage [W/kg]			
		(T0)	(T1)	Statistical parameters		(T0)	(T1)	Statistical parameters	
Variable	*n*	MD	MD	*p*-value	Effect size r	MD	MD	*p*-value	Effect size r
total	81	79.55	87.5	<.001	.38	1.12	1.22	<.001	.39
male	28	103.13	108.63	.124	.29	1.27	1.27	.118	.30
female	53	75	77.5	.002	.43	1.06	1.17	<.001	.45
Age group 60–64									
(m = 22%; w = 78%)	18	67.34	83.49	.058	.45	1.07	1.08	.071	.43
Age group 65–69									
(m = 29%; w = 71%)	42	84.72	92.09	.033	.33	1.19	1.27	.015	.38
Age group 70–74									
(m = 57%; w = 42%)	21	75	87.5	.042	.46	0.96	1.14	.070	.40

MD, median; p, significance <.01 via Wilcoxon-test; r, Pearson's r.

#### 3.2.2 SF-36

In terms of subjective perception of HRQoL, no gender or age differences were found at either the pre-test or post-test using the Mann–Whitney U test. Measured by the Physical Component Score (PCS), the group means of men and women were not significantly different (pre-test: *p* = .500; post-test: *p* = .913), nor were there any significant differences between the age groups (*p* > .05 for all).

Looking at the changes in group mean values before and after the exercise program, similar to the PWC 130, a significant difference (all *p* < .001) was found for the entire sample, for women with a medium effect, and even a strong effect for the age group 65–69 (see Table 2). Using the SF-36, no significant change in the subjective perception of health as a result of exercise was observed in the youngest age group 60–64 years (*p* = .039), age group 70–74 (*p* = .296) or in males (*p* = .084). The non-significant improvement in the 70–74 age group can be explained by the higher proportion of men in this subgroup.

**Table 2:** Results of the physical component score (PCS) from SF-36 baseline (T0) and post-test (T1).

Task		SF-36 PCS pre-test (T0)	SF-36 PCS post-test (T1)	Statistical parameters	Effect size
Variable	*n*	MD	MD	*p*-value	Pearson's r
Total	81	47.33	50.30	<.001	.44
Male	28	47.72	50.47	.084	.32
Female	53	47.12	50.22	<.001	.49
Age group 60–64 (m = 22%; w = 78%)	18	46.63	50.6	.039	.49
Age group 65–69 (m = 29%; w = 71%)	42	46.94	49.9	<.001	.51
Age group 70–74 (m = 57%; w = 42%)	21	48.67	50.9	.296	.23

MD, median; p, significance <.01 via Wilcoxon-test.

#### 3.2.3 PWC-130 & SF-36

The Spearman correlation showed no significant correlation between the subjective assessment of health and the objective assessment of the physical functional status of the participants. There is a discrepancy between these two parameters in both the pre-test (*p* = .149) and post-test (*p* = .321). Also, an analysis of the data in age- and gender-specific groups also revealed no significant correlations (all *p* > .08) between the PCS (SF-36) and relative performance (PWC-130).

In relation to the exercise program, there was also no significant correlation between the change in health perception (PCS score) and the physical performance of the participants. As a result, not all participants improved their total PCS score, which showed a better relative performance on the PWC-130 (total group: *p* = .601; age- and gender-related all *p* > .191).

## 4 Discussion

The present study evaluated the effects of a 24-week multidimensional exercise program on subjective well-being and cardiovascular fitness in previously inactive older adults, with primary emphasis on age- and gender-specific comparisons in the absence of a control group. The results show that the intervention led to statistically significant improvements in both subjective health (as measured by SF-36 PCS) and objective cardiovascular fitness (PWC-130). Significant improvements in subjective physical health (SF-36 PCS) were observed for the total sample, for women, and for participants aged 65–69. The 60–64 subgroup showed a moderate effect size (*r* = .49) but the *p*-value (*p* = .039) did not meet the adjusted significance threshold and should therefore be interpreted as a non-significant trend. Improvements in cardiovascular fitness (PWC-130) were robust for the total sample and for women, but age-specific PWC-130 improvements did not consistently reach *α* = .01 Notably, no significant correlation was found between improvements in cardiovascular fitness and perceived health status, suggesting that gains in subjective well-being and physiological performance may follow different pathways.

### 4.1 Subjective well-being

In line with the study goals, subjective well-being—measured by the physical component score (PCS) of the SF-36—showed significant improvement after the intervention for the total sample (*p* < .001, *r* = .44). This aligns with previous findings suggesting that multidimensional interventions have a moderate impact on health-related quality of life (HRQoL) in older adults (6, 7). Notably, however, the observed improvements in this study were primarily driven by female participants (*p* < .001, *r* = .49) and those aged 65–69 (*p* < .001, *r* = .51). These outcomes contrast with prior literature that reports stronger associations between physical activity and HRQoL in older men (9, 10).

One explanation could be the relatively high baseline PCS scores for both men (47.72) and women (47.12), exceeding normative reference values (M = 44.80), which may have limited observable room for improvement, particularly in men (38, 42). Additionally, men may possess a higher baseline level of self-confidence in physical performance, which could attenuate perceived gains in well-being following exercise (43).

The absence of significant change in the 70–74 age group (*p* = .296) is likely related to the disproportionately higher representation of men within this subgroup (57%), further supporting a gender-specific interpretation of these results. Nevertheless, even in this group, a non-significant trend toward improvement was observed.

### 4.2 Physical endurance performance

Cardiovascular fitness, assessed via the PWC-130, increased from pre- to post-test in all participants, confirming the general efficacy of the exercise program. However, significant improvements were observed only in the total sample (*p* < .001, *r* = .39), in women (*r* = .45), and in the 65–69 years age group with a moderate effect (*r* = .38). This may reflect gender-based differences in training response, as documented in earlier studies on concurrent training (CT) (17, 20). Indeed, older adults tend to show high variability in responsiveness to aerobic training (non-response rates between 1.4% and 63.4%), with women potentially benefiting more from concurrent training programs (17). The baseline parity in relative PWC-130 scores between men and women indicates that gender-specific effects may stem more from differences in adaptation rather than starting fitness levels.

Interestingly, all age groups (60–64, 65–69, and 70–74 years) showed a positive trend in cardiovascular fitness. However, significant improvements were observed only in the 65–69 years group for both absolute and relative performance, and in the absolute wattage of the 70–74 years group, where medium effect sizes were found (*r* ≈ .33–.46). These findings suggest that age alone does not preclude measurable improvements in cardiovascular fitness, which aligns with the results of some studies, though contradicting others that have found diminished effects in participants over 65 years (20). However, due to small sample sizes and high variability in their meta-analysis, these results should be interpreted with caution. The modest overall effect size might be due to the limited intensity and duration of the endurance components within the multidimensional framework, as well as the general heterogeneity in training responsiveness among older adults (2, 17, 18).

Notably, in this study, eight participants who were initially unable to complete the PWC-130 at baseline were able to do so after the program, indicating practical benefits of the training intervention even beyond statistical measures.

### 4.3 Relationship between subjective and objective outcomes

Despite the significant improvements in both subjective and objective health indicators, no significant correlation was found between changes in PCS and relative PWC-130 performance, neither at baseline nor post-intervention (all *p* > .14). These results contrast with previous research (6, 7), suggesting a potential disconnect between perceived and measured health benefits. This may be due to the complex interplay of psychosocial and physiological factors in older adults, including inter-individual variability in adaptation and baseline characteristics (6, 17). It raises the question of whether improvements in cardiovascular fitness alone are sufficient to enhance subjective well-being, or whether other psychosocial components must be addressed. In the future, a case-by-case analysis could be considered to determine which participants, based on their baseline characteristics, benefited most from the training intervention.

### 4.4 Limitations

While the results are encouraging, several limitations must be acknowledged. Most notably, the absence of a control group restricts the ability to attribute observed improvements solely to the intervention. Comparisons with inactive controls or those engaging in other training modalities were not possible. Furthermore, despite screening out regularly active individuals, varying levels of physical activity in daily life—such as walking, gardening, or occupational tasks—were not controlled for, possibly affecting inter-individual training responses. In addition, the use of convenience sampling may limit the representativeness of the findings, as older adults who voluntarily participated were likely motivated to initiate change, whereas those not seeking behavioral modification were not reached.

Variability in exercise intensity due to participants’ ability to opt out of difficult exercises and the flexible delivery of sessions (e.g., due to instructor illness or scheduling) may also have influenced results. Even with a minimum attendance requirement of 75%, the actual training load likely varied across participants. Lastly, the heterogeneity of older adult populations, both in this study and in the literature, poses a challenge for standardizing outcomes and drawing generalizable conclusions.

## 5 Conclusion

In summary, the 24-week multidimensional training intervention successfully improved both subjective physical health perception and cardiovascular fitness among inactive older adults. The greatest benefits were observed in women and individuals aged 65–69, both in subjective well-being and physical performance. Although improvements in cardiovascular fitness were seen in all groups, no significant changes in relative performance of PWC 130 and PCS of the SF-36 were found among men, in the youngest (60–64) and oldest age group (70–74), likely due to gender distribution and other moderating factors.

While the findings support the efficacy of such interventions in older populations, the absence of a correlation between subjective and objective measures suggests a need for further research. Future studies should incorporate control groups and more rigorous monitoring of activity levels and exercise intensity. Nevertheless, this program presents a promising foundation for gender- and age-specific health promotion in older adults.

## Data Availability

The raw data supporting the conclusions of this article will be made available by the authors, without undue reservation.
